# Efficacy and Mechanism of a Chinese Classic Prescription of Yueju in Treating Nonalcoholic Steatohepatitis and Protecting Hepatocytes from Apoptosis

**DOI:** 10.1155/2020/8888040

**Published:** 2020-10-29

**Authors:** Xiao-Li He, Yan-Ming He, Dan Zhang, Hong-Shan Li, Qiang Zhang, Sha-Sha Yuan, Zeng Zhang, Yan-Yan Wang, Cheng-Hao Liu, Chao-Hua Fan, Yun-Hao Li, Min Zheng, Hong-Jie Yang, Ping Zhou

**Affiliations:** ^1^Department of Endocrinology, Yueyang Hospital of Integrated Traditional Chinese and Western Medicine, Shanghai University of Traditional Chinese Medicine, Shanghai 200437, China; ^2^Department of Hepatology, Ningbo Huamei Hospital, University of Chinese Academy of Sciences, 41 Xibei Road, Ningbo 315010, China; ^3^State Key Laboratory of Molecular Engineering of Polymers, Department of Macromolecular Science, Fudan University, 220 Handan Road, Shanghai 200433, China

## Abstract

Yueju, a famous classic Chinese prescription, has been extensively used in treating depression syndromes for hundreds of years. Recent studies have reported that Yueju showed good effects in treating metabolic diseases, such as obesity and hyperlipidemia. Nonalcoholic steatohepatitis (NASH), which leads to cirrhosis and severe cardiovascular diseases, is closely linked to obesity and abnormal lipid metabolism. In this study, Yueju could decrease the levels of alanine aminotransferase, aspartate transaminase, triglyceride, cholesterol, and low-density lipoprotein-C but increase the high-density lipoprotein-C in the serum of the NASH rat model induced by high-fat and high-cholesterol diet. Yueju could alleviate hepatosteatosis by increasing the phosphorylation of acetyl-CoA carboxylase and inhibiting the expression of fatty acid synthase and stearoyl-CoA desaturase 1. Yueju downregulated the expression of *α*-smooth muscle actin and collagen type 1A1, ameliorating the liver fibrilization. Yueju could also protect the hepatocytes from apoptosis by upregulating antiapoptosis protein Bcl-2 and X-linked inhibitor of apoptosis protein and downregulating apoptotic proteins Bax and cleaved poly ADP-ribose polymerase. Thus, Yueju could improve liver function, regulate lipid metabolism, alleviate hepatosteatosis and fibrosis, and protect hepatocytes from apoptosis against NASH. Yueju may be used as an alternative effective medicine for NASH treatment.

## 1. Introduction

Nonalcoholic steatohepatitis (NASH) is a nonalcoholic fatty liver disease (NAFLD), which is associated with metabolic hepatitis. The global prevalence of NAFLD is currently estimated to be 25%, in which approximately 15% of all cases exhibit signs of NASH [1–3]. Contrary to nonhepatitis NALFD, NASH has more severe steatohepatitis and may cause fibrosis and cirrhosis, which are the key risk factors in the progression of hepatocellular carcinoma [4]. Given its close relation to obesity and other metabolic diseases, NASH has become one of the most common reasons for receiving a liver transplant in western countries [5]. The typical pathological changes in patients with NASH are hepatosteatosis, ballooning degeneration, infiltration of inflammatory cells, and spotty necrosis of the liver tissue [6]. Bland hepatosteatosis, which is a characteristic of the accumulation of excessive free fatty acids (FFAs) in the hepatocytes, can be triggered by the necroinflammatory response to induce NASH along with lipid peroxidation, inflammation, oxidative stress, and endoplasmic reticulum stress (ERS). This process is the so-called double-hit hypothesis [7]

Acetyl-CoA carboxylase (ACC) is a key rate-limiting enzyme in the synthesis of fatty acids, and the phosphorylation of ACC (p-ACC) can inhibit the production of fatty acids [8]. Fatty acid synthase (FASN) and stearoyl-CoA desaturase 1 (SCD1) are two crucial enzymes of the fat synthase system to promote the syntheses of long-chain saturated fatty acids and monounsaturated fatty acids, respectively [9, 10]. The accumulation of excessive FFAs can cause oxidative stress and chronic inflammation, inducing hepatocyte apoptosis in patients with NASH [11]. The B-cell lymphoma-2 (Bcl-2), which downregulates apoptosis, and Bcl2-associated X (Bax), which upregulates apoptosis, play important roles in the mitochondrion-mediated apoptosis [12, 13]. Irreparable DNA damage is another important indicator of cell apoptosis. The X-linked inhibitor of apoptosis protein (XIAP), one of the most effective IAP-related inhibitors, can prevent apoptosis induced by DNA damage [14]. The cleaved fragment of poly ADP-ribose polymerase 1 (PARP1) increases when DNA damage occurs [15]

Yueju prescription (YJP) is a famous classical prescription in traditional Chinese medicine and has been widely used for more than 700 years. This compound contains five herbal medicines, including Rhizoma Cyperi, Rhizoma Chuanxiong, Fructus Gardeniae, Rhizoma Atractylodis, and Massa Fermentata. Among these herbal medicines, Massa Fermentata is a mixture of six substances produced by fermentation, namely, *Semen armeniacae amarum, Semen phaseoli, Artemisiae annuae, Polygonum lapathifolium L.var. Sali-cidolium* Sibth*, Xanthium sibiricum* Patrin, and flours. Yueju is used to treat the depression syndrome, such as hepatic and damp depression syndromes [16]. Patients who are obese and with NAFLD often have depression symptoms, such as hypochondrium distension, fidgety, and irritancy. Yueju is recently reported to be used in treating metabolism-associated diseases. Clinical studies have shown that Yueju could reduce the fasting blood glucose (FBG), postprandial 2-hour blood glucose (P2HBG), hemoglobin A1C (HbA1C), triglyceride (TG), cholesterol (TC), and very-low-density lipoprotein (VLDL). This medicine could also improve the homeostasis model assessment-*β* (HOMA-*β*) index for patients with prestage of type 2 diabetes [17]. Yueju could reduce the FBG and P2HBG of patients with metabolic syndrome, in particular, those with obesity and abnormal glucose tolerance [18, 19]. In patients with hyperlipidemia, Yueju could decrease the TC, TG, and low-density lipoprotein-C (LDL-C) levels in the serum to improve lipid metabolism [20]. *In vivo* studies have shown that Yueju could lower the body weight, blood pressure, FBG, TG, TC, and LDL. In addition, this medicine can increase the high-density lipoprotein (HDL) in the serum of rats with metabolic syndrome by increasing the phosphorylation of adenosine 5ʹ-monophosphate-activated protein kinase-*α* [21].

Although Yueju could treat hyperglycemia and hyperlipidemia, no definite evidence that Yueju treats metabolic associated fat liver diseases is currently available. This study was performed to evaluate the efficacy of Yueju in treating NASH and elucidate the possible mechanism.

## 2. Materials and Methods

### 2.1. Animals

Forty-eight male Sprague-Dawley rats (6 weeks old; weight, 180–220 g) were purchased from the SLAC Laboratory Animal Co., Ltd. (Shanghai, China) and adaptively fed for one week in the Animal Experimental Center of Ningbo University (License No. SYXK2019-0005). Then, the rats were randomly assigned to the normal group (*n* = 8) and high-fat and high-cholesterol diet (HFHCD) group (*n* = 40), which were fed with a standard chow diet and an HFHCD, respectively, for 14 weeks. The HFHCD, which contained 10% lard, 2% cholesterol, and 88% basic feed (AIN-93M diet), was used to induce the NASH model as previously described [22]. The standard chow diet was produced as the formula of AIN-93M with 4% fat.

All experiments were approved (ID: 2019-243) by the Experimental Animal Ethics Committee of Ningbo University and conducted in accordance with the *Guide for the Care and Use of Laboratory Animals* (National Institute of Health, NIH Publication 86–23, revised 1996).

### 2.2. Diets and Drugs

HFHCD and standard chow diet were produced by Jiangsu Xietong Bioengineering Co. Ltd. The crude herbal medicines of Yueju prescription (Batch number 181018) were purchased from the Anhui Xinxing Chinese Herbal Pieces Co. Ltd. (Bozhou, China). The Yueju extract powder was prepared by the authors. Rosiglitazone (RSG) (Lot. H20030569), which is a peroxisome proliferator-activated receptor-*γ* (PPAR*γ*) agonist used as a positive drug *in vivo* [23], was purchased from Chengdu Hengrui Pharmaceutical Co. Ltd. Fenofibrate (FNF) (Lot. S5812), which is a PPAR*α* agonist used as a positive drug *in vitro* [24], was purchased from Selleck Chem, Inc., (Shanghai, China).

### 2.3. Model Building

The HFHCD group was fed with HFHCD for 14 weeks to build the NASH model *in vivo* as previously described [25]. The HepG2 cells were treated with 300 mM or 2 mM FFAs (oleic acid: palmitic acid = 2 : 1 ratio, dissolved by isopropanol) to build the hepatosteatosis model or apoptotic hepatocytes model *in vitro* as previously described [26].

### 2.4. Group and Treatment

The rats in the HFHCD group were distributed randomly into the model groups of YJP0.2, YJP1, YJP5, and RSG (*n* = 8 in each group) after they were fed for 10 weeks. The YJP0.2, YJP1, YJP5, and RSG groups were treated with 0.2, 1, and 5 g/kg YJP extract and 0.4 mg/kg RSG, respectively, from the first day of the 11th week.

### 2.5. Components and Extraction Method

Yueju contains Rhizoma Cyperi, Rhizoma Chuanxiong, Fructus Gardeniae, Rhizoma Atractylodis, and Massa Fermentata. The crude herbal medicines were dried, chopped into pieces, and decocted with 10 times weight of water at 100°C for 1 hr, and then the supernatant was collected after the dregs were filtered. The dregs were again processed similarly. Two supernatants were collected together and distilled in a 75°C water bath for 2 hr. The concentrated solution was lyophilized at −80°C in a low-temperature vacuum drying oven (Labconco® FreeZone® Plus™ 12 L, Labconco Corp. MO, USA) for 48 hr to obtain Yueju powder.

### 2.6. Reagents

Fetal bovine serum (FBS), RPMI 1640 medium, minimum Eagle's medium (MEM), sodium pyruvate (SP), nonessential amino acid (NEAA), glutamine (Gln), penicillin–streptomycin solution, 0.25% trypsin with ethylenediaminetetraacetic acid (EDTA), and 0.25% trypsin without EDTA were purchased from Gibco (Thermo Fisher Scientific, Inc. MA, USA). Insulin-transferrin-sodium selenite medium supplement (ITS), dexamethasone (DXM), dimethyl sulfoxide (DMSO), oleic acid, palmitic acid, and isopropanol were purchased from Sigma-Aldrich, LLC. (MUC, GER). Phosphate-buffered saline (PBS) was purchased from Hyclone (Thermo Fisher Scientific, Inc. MA, USA). Cell counting kit-8 (CCK-8) was bought from Dojindo, Inc. (Shanghai, China). The following kits were purchased from Jiancheng Bioengineering Institute (Nanjing, Jiangsu, China): test kits for serum alanine aminotransferase (ALT), aspartate transaminase (AST), TG, TC, HDL-C, and LDL-C; oil red O staining kit; Masson staining kit; and hematoxylin-eosin (HE) staining kit. The Liver TG test kit was purchased from Dongou Diagnostic Products Co. Ltd. (Wenzhou, Zhejiang, China). Terminal deoxynucleotidyl transferase-mediated dUTP nick-end labeling (TUNEL) kit was purchased from Beyotime Biotechnology, Inc. (Shanghai, China).

### 2.7. Cell Culture

The human hepatic cell line (HepG2) was obtained from the Institute of Liver Disease, Shanghai University of Traditional Chinese Medicine. HepG2 cells were cultured with 10% FBS, 1% SP, 1% NEAA, 1% Gln, and MEM in 5% CO_2_ at 37°C. The cells were passaged every 2 days when the density was 80%.

### 2.8. Cell Treatment

HepG2 cells were cultured on 6-well plates or 6 cm culture dishes with a density of 2 × 10^6^ cells/well or 4 × 10^6^ cells/dish for 24 h. Then, HepG2 was incubated with FFAs, YJP (dissolved by ultrapure water and filtered by 4 *μ*M Millex filter first), and FNF, respectively, for 24 hr. Then, the cell lysates of each group were collected and stored at −70°C in a refrigerator.

### 2.9. Assessment of the Cytotoxicity of Yueju Prescription and FFAs to HepG2 with CCK-8

The HepG2 cells were incubated with 0, 12.5, 25.0, 50.0, 100.0, 200.0, 300.0, 400.0, and 500.0 *μ*g/mL of YJP for 24 hr. HepG2 cells were also incubated with 0, 100.0, 200.0, 300.0, 400.0, 600.0, 800.0, 1000.0, 1500.0, and 2000.0 *μ*M of FFAs for 24 hr. Cytotoxicity was measured by using the CCK-8 kit according to the manufacturer's protocol described.

### 2.10. RNA Isolation, cDNA Synthesis, and Real-Time Polymerase Chain Reaction (PCR)

Isolation and collection of total RNA, reversion of transcription of RNA, and real-time PCR were conducted using previously described methods [27]. The quantitative results for fluorescence spectroscopy were calculated via 2^ − ΔΔCt^ by using the normalization method. The primer sequences of mRNA are listed in Table S1 of the Supplemental Material.

### 2.11. Western Blot Analysis

Cell lysates were prepared and Western blot was performed by methods as previously described [28]. The gray value was analyzed, and the relative expression level of protein was obtained via Image J 1.51 software (NIH Image, Bethesda, MD, USA). The primary and secondary antibodies used are listed in Table S2 of the Supplemental Material.

### 2.12. Oil Red O Staining

HepG2 was cultured and treated in 24-well plates. After the media were discarded, the cells were fixed with 4% paraformaldehyde and dyed with oil red O working solution to mark the lipid droplets. Then, the cells were stained with hematoxylin for 1-2 min to visualize the nuclei. After covering with glycerin gelatin, the cells were observed using an inverted microscope.

### 2.13. HE Staining

The liver tissues were dehydrated through a serial alcohol gradient and embedded in paraffin wax blocks. Before staining, 4 *μ*m thick liver tissue sections were dewaxed in xylene, rehydrated by decreasing concentrations of ethanol, and washed in PBS. Then, sections were stained with HE. After staining, the samples were dehydrated by using increasing concentrations of ethanol and xylene, and then the samples were covered with neutral resin. The NAFLD activity score (NAS) was determined as follows [29]: steatosis (0, <5%; 1, 5%–33%; 2, 33%–66%; and 3, >66%); intralobular inflammation (0, no lesions; 1, <2 lesions/field of view; 2, 2–4 lesions/field of view; and 3, >4 lesions/field of view); and ballooning degeneration (0, none; 1, rare new balloon cells; and 2, common new balloon cells).

### 2.14. Masson Staining

Liver paraffin sections were dewaxed, rehydrated, and washed in PBS as HE staining. Then, the sections were stained with Weigert's iron hematoxylin, ponceau-fuchsin, and aniline blue. Blue stained areas represented collagen fibers. Positive areas were semianalyzed via Image J 1.51 software (NIH Image, Bethesda, MD, USA).

#### 2.14.1. Triglyceride Test for Liver Tissue

Liver tissues were collected from the same position of each liver and ground into homogenate with acetone–ethanol solution (1 : 1, v/v). After centrifugation, the supernatant was aspirated into a 1.5 mL EP tube. Then, the contents of TG were measured by using a TG test kit as the manufacturer's protocol described.

#### 2.14.2. Triglyceride Test for the Cell

After being washed with cold PBS twice, the cells were filled with 1 mL of PBS per 5 − 10 × 10^6^ cells and smashed with an ultrasonic wave at 200 W for 3 s three times. Then, the cell lysates were transferred to a 1.5 mL EP tube. The TG contents were measured by using a TG test kit according to the manufacturer's protocols.

### 2.15. Serum AST, ALT, TG, TC, HDL, and LDL Test

Serum levels of AST, ALT, TG, TC, HDL-C, and LDL-C were measured using test kits (Nanjing Jiancheng Bioengineering Institute, Nanjing, China) as described by the manufacturer's protocols.

### 2.16. Immunohistochemical Staining

Liver paraffin sections were dewaxed and rehydrated first. After the antigen repair and elimination of endogenous peroxidase, 10% goat serum was added to the sections for 30 min, and the sections were subsequently covered with 1 *μ*g/mL *α*-smooth muscle actin (*α*-SMA) primary antibody (see Table S2) at 4°C for 12 hr. Horseradish peroxidase (HRP) solution was added to conjugate the primary antibody, while 2% diaminobenzidine (DAB) solution was used to visualize the positive area. The sections were stained with hematoxylin dye to visualize the nuclei. Positively stained areas were semianalyzed via Image J 1.51 software (NIH Image, Bethesda, MD, USA).

### 2.17. TUNEL Staining

Liver paraffin sections were dewaxed and rehydrated first. The sections were treated with proteinase K followed by incubation with the TdT reaction mixture and then incubated in streptavidin-HRP. The color was developed with DAB as a chromogen. Apoptotic nuclei exhibiting fragmented DNA were stained dark brown.

### 2.18. Annexin V-FITC/PI Staining

After treatment with YJP and FNF for 24 hr, the cells were digested by 0.25% trypsin, washed by cold PBS twice, and resuspended by 1× binding buffer into 1 × 10^5^ cells/mL. The cells were then stained by annexin V-FITC and PI dye for 15 min at room temperature as described by the product protocol, detected by flow cytometry, and analyzed by WinMDI 2.9 software (Purdue University Cytometry Laboratories, IN, USA).

### 2.19. Statistical Analysis

Data were expressed as mean ± standard deviation. Statistical analyses were operated using one-way ANOVA and least significant difference test, and *P* < 0.05 was considered statistically significant.

## 3. Results

### 3.1. Yueju Could Improve the Liver Appearance and Function

**Figure 1:**
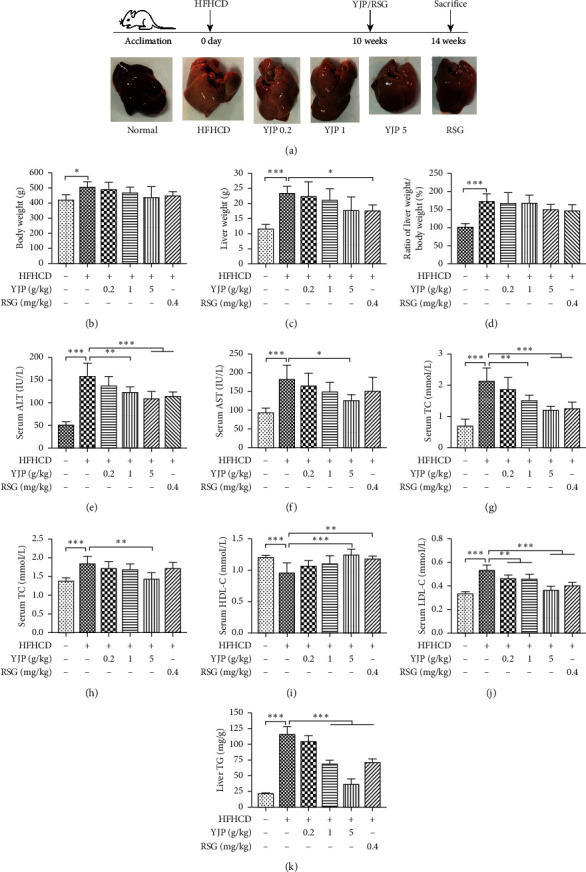
Yueju effects on basal information and biochemical profile in treating rats fed with HFHCD. (a) Experimental processes and liver images. (b) Body weight. (c) Liver weight. (d) The ratio of liver weight and body weight. (e) Serum ALT. (f) Serum AST. (g) Serum TG. (h) Serum TC. (i) Serum HDL-C. (j) Serum LDL-C. (k) The TG content of liver tissue. Data were expressed as means ± standard deviation (*n* = 8). Compared with the model group, symbols ^*∗*^,^*∗∗*^, and ^*∗∗∗*^ represent the significance at *P*  <  0.05, *P*  <  0.01, and *P*  <  0.001, respectively.

*In vivo*, the body and liver weights of rats from the model group were significantly higher than those from the normal control group (see Figure 1(b)). Compared with those in the model group, the body and liver/body ratios of rats from the YJP0.2, YJP1, YJP5, and RSG groups were reduced gradually but without statistical differences (see Figures 1(b) and 1(d)). After treatment with 5 g/kg YJP or 0.4 mg/kg RSG for 4 weeks, the appearance of the livers became natural, and swelling was decreased compared with those of the model group (see Figure 1(a)).

On the liver function, the serum ALT was lowered significantly after treatment with 1 and 5 g/kg YJP, and 0.4 mg/kg RSG, respectively (see Figure 1(e)). The serum AST was decreased significantly after treatment with 5 g/kg YJP (see Figure 1(f)). Thus, YJP could improve the liver appearance and function of the NASH model rats.

### 3.2. Yueju Could Alleviate Hepatosteatosis *In Vivo* and *In Vitro*

**Figure 2:**
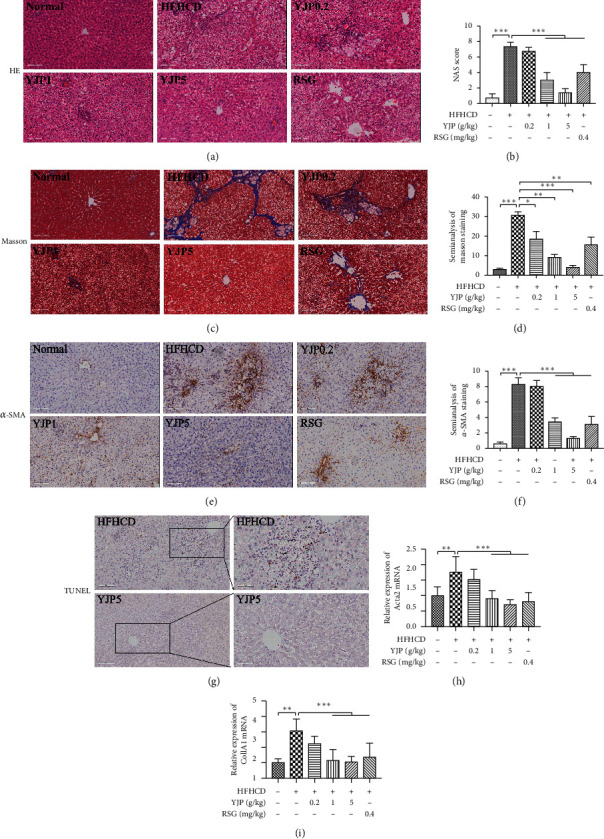
Yueju effects on ameliorating the hepatosteatosis and liver fibrosis *in vivo*. (a) HE staining, magnification: 200×. (b) The statistical analyses of NAS score. (c) Masson staining, magnification: 200×. (d) The semianalyses of positive area which was stained blue in Masson stained liver tissue sections. (e) The immunohistochemical staining of *α*-SMA, magnification: 200×. (f) The semianalyses of positive area which was stained brown by *α*-SMA immunohistochemical staining. (g) TUNEL staining, magnification: 200× (left) and 400× (right). Apoptotic hepatocytes (black arrow). (h) The mRNA expression of *Acta2*. (i) The mRNA expression of *Col1A1*. Data were expressed as means ± standard deviation (*n* = 8). Compared with the model group, symbols ^*∗*^,^*∗∗*^, and ^*∗∗∗*^ represent the significance at *P*  <  0.05, *P*  <  0.01, and *P*  <  0.001, respectively.

Compared with those in the model group, the NAS scores, liver TG, and serum TG were decreased significantly after treatment with 1 and 5 g/kg YJP and 0.4 mg/kg RSG, respectively (see Figures 2(a), 2(b), 1(k), and 1(g)). The serum TC was reduced significantly after treatment with 5 g/kg YJP (see Figure 1(h)). The serum HDL-C was increased significantly after treatment with 5 g/kg YJP and 0.4 mg/kg RSG (see Figure 1(i)). The serum LDL-C was decreased significantly after treatment with 0.2, 1, and 5 g/kg YJP and 0.4 mg/kg RSG (see Figure 1(j)).

**Figure 3:**
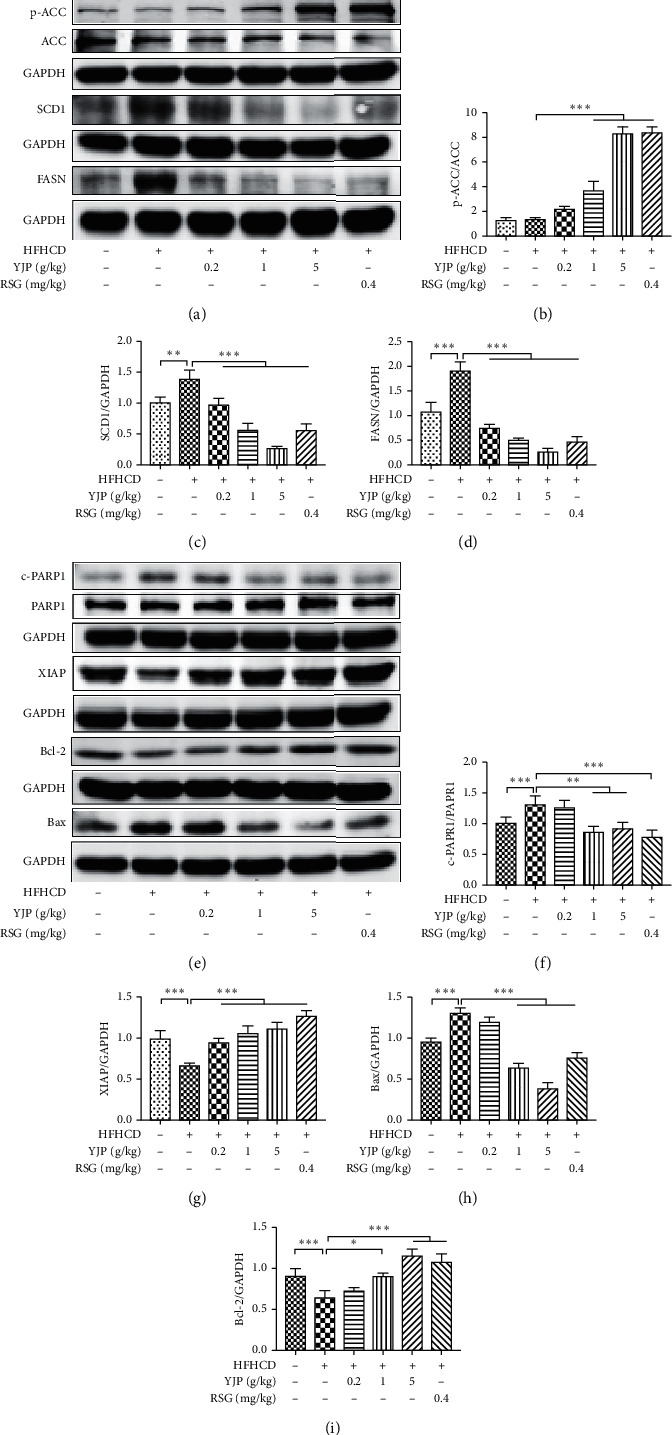
Yueju effects on improving the protein expression of lipid metabolism and apoptosis associated cytokines *in vivo*. (a) The protein expression of p-ACC, ACC, FASN, and SCD1 detected by Western blot. (b–d) The gray value analyses of *p*-ACC/ACC, FASN/GAPDH, and SCD1/GAPDH. (e) The protein expression of c-PARP1, PARP1, XIAP, Bcl-2, and Bax detected by Western blot. (f–i) The gray value analyses of c-PARP1/PARP1, XIAP/GAPDH, Bcl-2/GAPDH, and Bax/GAPDH. Data were expressed as means ± standard deviation (*n* = 8). Compared with the model group, symbols ^*∗*^,^*∗∗*^, and ^*∗∗∗*^ represent the significance at *P*  <  0.05, *P*  <  0.01, and *P*  <  0.001, respectively.

The Western blot results showed that the ratio of p-ACC/ACC was significantly elevated after treatment with 1 and 5 g/kg YJP and 0.4 mg/kg RSG compared with that in the model group (see Figures 3(a) and 3(b)). FASN and SCD1 were downregulated after treatment with 0.2, 1, and 5 g/kg YJP and 0.4 mg/kg RSG compared with those in the model group (see Figures 3(a), 3(c), and 3(d)).

**Figure 4:**
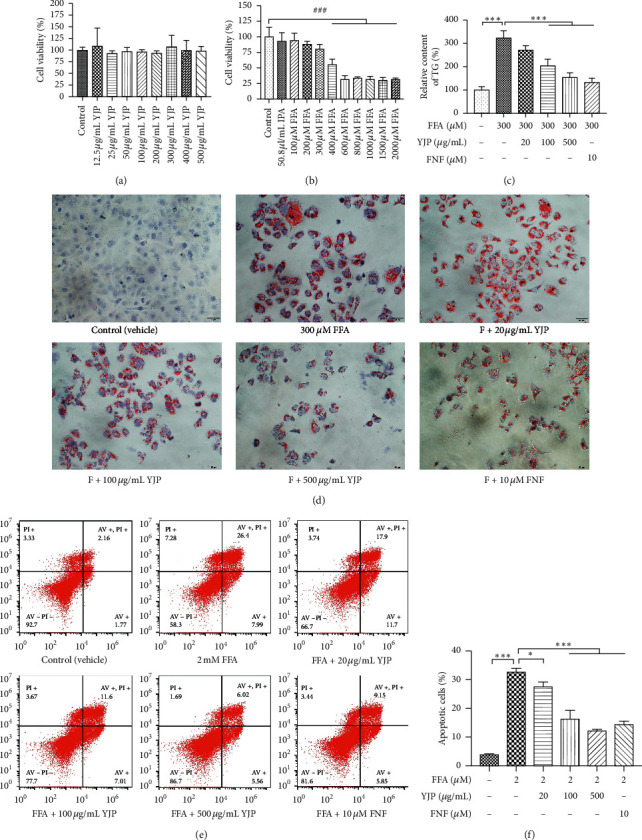
Yueju effects on ameliorating the hepatosteatosis and apoptosis in HepG2. (a) The cell viability of HepG2 treated with YJP ranging from 0 to 500 *μ*g/mL. (b) The cell viability of HepG2 treated with FFAs (0, 100, 200, 300, 400, 600, 800, 1000, 1500, and 2000 *μ*M). Compared with the control group, the symbol ^###^ represents the significance at *P*  <  0.001. (c) The TG content of HepG2 treated with different doses of YJP. (d) The oil red O staining of HepG2 treated with different doses of YJP, magnification: 200×. (e) and (f) The percentage of apoptotic HepG2, which was induced by FFAs, treated with YJP and FNF, and stained with annexin V-FITC/PI dye, was analyzed by flow cytometry. Data were expressed as means ± standard deviation. Compared with the model group, symbols ^*∗*^ and ^*∗∗∗*^ represent the significance at *P*  <  0.05 and *P*  <  0.01. Control group was cultured with normal culture medium. IPA group was cultured with normal culture medium containing 50.8 *μ*l/mL isopropanol.

The cytotoxicity of YJP (0, 12.5, 25, 50, 100, 200, 300, 400, and 500 *μ*g/mL) on the HepG2 cell line was tested by using the CCK-8 kit *in vitro*. After treatment for 24 hr, the cell viability was not significantly decreased (see Figure 4(a)). Three concentrations of YJP (20, 100, and 500 *μ*g/mL) were selected to evaluate its efficacy. Then, the cytotoxicity of the FFAs (0, 100, 200, 300, 400, 600, 800, 1000, 1500, and 2000 *μ*M) on HepG2 was tested by using the CCK-8 kit. The cell viability was significantly decreased when the FFAs' concentration was over 400 *μ*M (see Figure 4(b)). Thus, 300 *μ*M FFAs were used to build the hepatosteatosis model *in vitro*. The results of oil red O staining showed that the lipid droplets were reduced significantly after treatment with 100 and 500 *μ*g/mL YJP and 10 *μ*M FNF (see Figure 4(d)). The relative contents of TG in HepG2 were significantly lowered after treatment with 100 and 500 *μ*g/mL YJP and 10 *μ*M FNF (see Figure 4(c)). These results showed that YJP could regulate lipid metabolism and, therefore, alleviate hepatosteatosis.

### 3.3. Yueju Could Improve the Liver Fibrosis in NASH Model Rats

Masson staining was employed to evaluate the fibrotic stage in NASH model rats and blue stained areas represented the collagen fibers. The collagen deposition and cross-linking of fibers were markedly heavier in the model group while the fibrotic stage was decreased after treatment with 1 and 5 g/kg YJP and 0.4 mg/kg RSG (see Figure 2(b)). Compared with that in the model group, the expression of fibrogenic genes, such as *Acta2* (gene name of *α*-SMA) and collagen type 1A (*Col1A1*), was downregulated significantly after treatment with 1 and 5 g/kg YJP and 0.4 mg/kg RSG (see Figures 2(h) and 2(i)). In the immunohistochemical staining, the expression of *α*-SMA was also downregulated after treatment with 1 and 5 g/kg YJP (see Figures 2(e) and 2(f)). Thus, YJP could decrease the fibrogenic markers (*α*-SMA and Col1A1) to ameliorate fibrosis.

### 3.4. Yueju Could Protect the Hepatocytes from Apoptosis *In Vivo* and *In Vitro*

The apoptosis of hepatocytes in the liver tissue was detected *in vivo* by using TUNEL staining. Numerous apoptotic hepatocytes were found in or around the necrosis areas after feeding with HFHCD for 14 weeks (see Figure 2(g), black arrows). Compared with the model group, the numbers of apoptotic hepatocytes were significantly decreased after treatment with 5 g/kg YJP (see Figure 2(g)).

Additionally, apoptosis-related proteins were detected to evaluate the protective effects of hepatocytes by YJP in the NASH model rats. Compared with that in the model group, the protein expression of XIAP and Bcl-2 was upregulated significantly in the rats treated with 1 and 5 g/kg YJP and 0.4 mg/kg RSG, respectively (see Figures 3(e), 3(g), and 3(i)). The protein expression of Bax and cleaved-PARP1 was downregulated significantly after treatment with 1 and 5 g/kg YJP and 0.4 mg/kg RSG (see Figures 3(e), 3(f), and 3(h)).

Given that the cell viability was significantly decreased when the concentration of FFAs was over 400 *μ*M (see Figure 4(b)), 2 mM FFAs were used to imitate the cytotoxicity of FFAs *in vitro* as previously described [30]. The apoptosis of the HepG2 cells was induced by 2 mM FFAs and treatment with 20, 100, and 500 *μ*g/mL YJP and 10 *μ*M FNF for 24 hr, respectively. Then, the cells were stained with annexin V-FITC/PI and analyzed by flow cytometry (DxFLEX, Beckman Coulter Inc, Atlanta, Georgia, USA) to evaluate the percentage of apoptotic cells. Compared with those in the model group, the percentage of early and late apoptotic cells was decreased significantly after treatment with 20, 100, and 500 *μ*g/mL YJP and 10 *μ*M FNF for 24 hr (see Figures 4(e) and 4(f)). These results indicated that YJP could protect the hepatocytes from apoptosis by upregulating Bcl-2 and XIAP and downregulating Bax and c-PARP1.

## 4. Discussion

Hepatosteatosis is the main pathological manifestation of patients with NASH. In the liver tissues, excessive FFAs are accumulated, esterified into TG, and stored in the hepatocytes as lipid droplets. Excessive lipid droplets overloaded in nonadipose cells, such as hepatocytes, lead to cell dysfunction and apoptotic death, triggering liver injury and fibrosis.

During the formation of hepatosteatosis, excessive accumulation of lipids is a core event along with dysfunction of synthesis, utilization, and transferring of FFAs. In addition to synthetases of FFAs, such as p-ACC, FASN, and SCD1, some key enzymes or signal transductors also play important roles in hepatosteatosis. PPAR*γ* can redirect FFAs into adipose tissues which have a unique capacity to store large amounts of FFAs [31]. Leptin and PPAR*α* can increase the utilization of FFAs in the mitochondria through the *β*-oxidation pathway [32, 33]. Moreover, FFAs can be assembled into the form of lipoproteins and secreted out of cells [34]. For Yueju, several active components from this medicine were reported to be effective in regulating these targets. Ligustrazine, which is an active component from Rhizoma Chuanxiong, could directly stimulate PPAR*γ* by ligand activation to suppress the function of hepatic stellate cells in liver fibrosis [35]. Geniposide, an active component of Fructus Gardeniae, could enhance leptin signaling to attenuate the level of A*β*1–42 in Alzheimer's disease and activate PPAR*γ* to reduce the blood glucose level in type 2 diabetes [36, 37]. Thus, Yueju might ameliorate hepatosteatosis by regulating these key signaling pathways. Then, the lipid metabolism and transfer-related signaling pathways will be detected to elucidate the mechanisms of Yueju in antihepatosteatosis.

Chronic inflammation is another crucial factor that leads to liver injury and fibrosis in patients with NASH [38]. Inflammation could influence lipid metabolism, cell apoptosis, and accumulation of extracellular matrix by regulating proinflammatory cytokines, polarizing macrophage, and activating T cell [39]. Approximately 15% of patients with NAFLD have fatty liver accompanied by steatohepatitis. Compared with nonhepatitis NAFLD, patients with NASH have a higher risk of hepatic fibrosis, HCC, cardiovascular diseases, type 2 diabetes, and other complications [40]. In this study, histopathological results showed that Yueju could decrease the infiltration of inflammatory cells to improve liver function and fibrotic stage. In the author's future studies, the proinflammatory cytokines, such as tumor necrosis factor-*α*, interleukin-6, and inflammatory cells, such as macrophages, will be explored to elucidate the anti-inflammation mechanism of Yueju.

In patients with NASH, hepatocyte apoptosis is often observed and closely related to the degree of hepatic fibrosis [41]. Excessive accumulation of lipids can induce the transition of the mitochondria membrane permeability and the release of cytochrome C to cause cell death [42, 43]. In endogenous apoptosis, the ERS is an independent and crucial factor [44]. Recent studies have shown that HFHCD caused ERS by regulating the C/EBP homologous protein (CHOP) and the phosphorylation of JNK to induce cell apoptosis [45, 46]. In this study, Yueju protected the hepatocytes from apoptosis by upregulating antiapoptosis proteins, such as Bcl-2 and XIAP, and downregulating proapoptosis proteins, such as Bax and c-PARP1. Whether Yueju could interfere the ERS is unclear. Hence, in further studies, the ERS-related pathways will be studied to reveal the mechanism of hepatocyte protection by Yueju.

Although this study showed that Yueju prescription could treat NASH, the specific active components of Yueju prescription remain unclear, and the mechanisms of Yueju prescription in treating NASH are still not elucidated. Thus, additional studies on these concerns will be conducted.

## 5. Conclusions

Yueju showed a novel efficacy in ameliorating hepatosteatosis. This traditional Chinese medicine could regulate lipid metabolism, improve hepatic fibrotic stages, and protect hepatocytes from apoptosis. Yueju may be a potential drug to treat metabolic associated fatty liver diseases and warrants further studies.

## Data Availability

The datasets used and analyzed during the current study are available from the corresponding author upon reasonable request.

## Supplementary Materials

Supplementary Materials Table S1: the primer sequences of mRNA used in this study. Table S2: information about manufacturers, species, lot. numbers, and concentrations of primary and secondary antibodies.
