# Supplementary material for: Efficacy and Mechanism of a Chinese Classic Prescription of Yueju in Treating Nonalcoholic Steatohepatitis and Protecting Hepatocytes from Apoptosis
Source: Evid Based Complement Alternat Med. 2020 Oct 29;2020:8888040. doi: 10.1155/2020/8888040 (PMC7644301; doi:10.1155/2020/8888040)
Supplement: Supplementary Materials — Table S1: the primer sequences of mRNA used in this study. Table S2: information about manufacturers, species, lot. numbers, and concentrations of primary and secondary antibodies. [file 8888040.f1.doc]

**Supplemental materials**

**Efficacy and mechanism of a Chinese classic prescription of Yueju in treating non-alcoholic steatohepatitis and protecting hepatocytes from apoptosis**

Xiao-Li He^1^, Yan-Ming He^1^, Dan Zhang^1^, Hong-shan Li^3^, Qiang Zhang^1^, Sha-Sha Yuan^1^, Zeng Zhang^1^, Yan-Yan Wang^1^, Cheng-Hao Liu^1^, Chao-Hua Fan^1^, Yun-Hao Li^1^, Min Zheng^1^, Hong-Jie Yang^1*^, and Ping Zhou^2*^

*^1^Department of Endocrinology, Yueyang Hospital of Integrated Traditional Chinese and Western Medicine, Shanghai University of Traditional Chinese Medicine, Shanghai, 200437, China*

*^2^State Key Laboratory of Molecular Engineering of Polymers, Department of Macromolecular Science, Fudan University, 220 Handan Road, Shanghai, 200433, China*

*^3^Department of Hepatology, Ningbo Huamei Hospital, University of Chinese Academy of Sciences, 41 Xibei Road, Ningbo, 315010, China*

^*^Corresponding author.

^**^Correspondence should be addressed to Ping Zhou; [pingzhou@fudan.edu.cn](mailto:pingzhou@fudan.edu.cn) or Hong-jie Yang; yanghongjie@shyueyanghospital.com.

**Table S1. Real-time PCR primer sequences.**

| Primers | Forward | Reverse |
| --- | --- | --- |
| α-SMA | 5'-GGAGATGGCGTGACTCACAA-3' | 5'-CGCTCAGCAGTAGTCACGAA-3' |
| Col1A1 | 5'-CATAAAGGGTCATCGTGGC-3' | 5'-TCAGGCTCTTGAGGGTAGTGT-3' |
| GAPDH | 5'-GGGGCTCTCTGCTCCTCCCTG-3' | 5'-AGGTGAGCCCCAGCCTTCTCC-3' |

**Table S2. The list of primary antibodies and secondary antibodies.**

|  | Antibodies | Anti-species | Inc. | Dilution | Lot. |
| --- | --- | --- | --- | --- | --- |
| Primary antibodies | Bcl-2 | Rabbit | Abcam | 1: 500 | Ab59348 |
|  | Bax | Rabbit | Abcam | 1: 1000 | Ab32503 |
|  | XIAP | Rabbit | Abcam | 1: 1000 | Ab2541 |
|  | c-PARP1 | Rabbit | Abcam | 1: 1000 | Ab32064 |
|  | PARP1 | Rabbit | Abcam | 1: 1000 | Ab32138 |
|  | P-ACC | Rabbit | CST | 1: 1000 | # 11818S |
|  | ACC | Rabbit | CST | 1: 1000 | # 3676S |
|  | SCD1 | Rabbit | Abcam | 1: 1000 | Ab236868 |
|  | FASN | Rabbit | CST | 1: 1000 | # 3180S |
|  | GAPDH | Mouse | Proteintech | 1: 5000 | 60004-1-Ig |
| Secondary antibodies | Anti-rabbit (680) | Goat | CST | 1: 10000 | # 5366 |
|  | Anti-mouse (800) | Goat | CST | 1: 10000 | # 5257 |
